# Development of the transcallosal motor fiber from the corticospinal tract in the human brain: diffusion tensor imaging study

**DOI:** 10.3389/fnhum.2014.00153

**Published:** 2014-03-18

**Authors:** Hyeok Gyu Kwon, Su Min Son, Sung Ho Jang

**Affiliations:** Department of Physical Medicine and Rehabilitation, College of Medicine, Yeungnam UniversityDaegu, South Korea

**Keywords:** transcallosal motor fiber, development, interhemispheric inhibition, corpus callosum, corticospinal tract

## Abstract

Transcallosal motor fiber (TCMF) plays a role in interhemispheric inhibition (IHI) between two primary motor cortices. IHI has been an important concept in development of the motor system of the brain. Many studies have focused on the research of the topography of TCMF, however, little is known about development of TCMF. In the current study, we attempted to investigate development of TCMF from the corticospinal tract (CST) in the human brain using diffusion tensor tractography. A total of 76 healthy subjects were recruited for this study. We reconstructed the TCMF, which was derived from the CST, by selection of two regions of interest below the corpus callosum (upper and middle pons). Termination criteria used for fiber tracking were fractional anisotropy <0.2 and three tract turning angles of <45, 60, and 75^°^. The subjects were classified into four groups according to age: group A (0–5 years), group B (6–10 years), group C (11–15 years), and group D (16–20 years). Significant differences in the incidence of TCMF were observed between group B and group C, and between group B and group D, with tract turning angles of 60 and 75^°^ (*p *< 0.05). However, no significant differences in any tract turning angle were observed between group C and group D (*p* > 0.05). In addition, in terms of the incidence of TCMF, no significant differences were observed between the three tract turning angles (*p* > 0.05). We obtained visualized TCMF from the CST with development and found that the incidence of TCMF differed significantly around the approximate age of 10 years. As a result, we demonstrated structural evidence for development of TCMF in the human brain.

## INTRODUCTION

Transcallosal motor fiber (TCMF), which indicates the connection fibers of the corpus callosum between primary motor cortices of the two hemispheres, plays a role in interhemispheric inhibition (IHI) between the two primary motor cortices (Netz, 1999; De Gennaro et al., 2004; Lenzi et al., 2007; Wahl et al., 2007; Wahl and Ziemann, 2008; Jang et al., 2009). IHI has been an important concept in development of the motor system of the brain. Associated movements or mirror movements are involuntary movements corresponding to voluntary movement on opposite sides of the body (Connolly and Stratton, 1968; Mayston et al., 1999). They are known to decrease with development of IHI (Connolly and Stratton, 1968; Wolff et al., 1983; Lazarus and Todor, 1987; Heinen et al., 1998; Mayston et al., 1999). In adult, associated movement is regarded as a pathological phenomenon (Connolly and Stratton, 1968). However, normally developing children and adolescents can represent the associated movements to a various degree depending on the motor task, development of the corpus callosum and maturation of motor control network (Mayston et al., 1999; Hoy et al., 2004; Shim et al., 2008; Koerte et al., 2009; Gasser et al., 2010; Qiu et al., 2010). On the other hand, decrement of IHI following brain injury is a basic mechanism of the contribution of the unaffected motor cortex, which has been regarded as a mechanism for motor recovery (Liepert et al., 2000; Manganotti et al., 2002; Shimizu et al., 2002; Jang et al., 2009; Jang, 2010). Therefore, clarification of the characteristics of IHI and TCMF has been an important topic in neuroscience.

Many previous studies have reported on IHI (Cohen et al., 1967; Connolly and Stratton, 1968; Wolff et al., 1983; Lazarus and Todor, 1987; Mayston et al., 1997, 1999; Müller et al., 1997; Heinen et al., 1998; Hamzei et al., 2002; Stefanovic et al., 2004; Manson et al., 2006; Koerte et al., 2009; Gasser et al., 2010; Koerte et al., 2010). Behavior study, electromyography, transcranial magnetic stimulation (TMS), and functional magnetic resonance imaging were used in most of these studies (Cohen et al., 1967; Connolly and Stratton, 1968; Wolff et al., 1983; Lazarus and Todor, 1987; Mayston et al., 1997, 1999; Müller et al., 1997; Heinen et al., 1998; Hamzei et al., 2002; Stefanovic et al., 2004; Manson et al., 2006; Koerte et al., 2009; Gasser et al., 2010; Koerte et al., 2010). However, these methods had a common limitation in that visualization and reconstruction of the neural tract, such as TCMF, could not be achieved. By contrast, recently developed diffusion tensor tractography (DTT), derived from diffusion tensor imaging (DTI), has a unique advantage in three-dimensional reconstruction of neural tracts by detecting the water diffusion properties (Kunimatsu et al., 2004). Several studies using DTI have reported on TCMF in normal subjects and in patients with brain injury (Wahl et al., 2007; Jang et al., 2009; Koerte et al., 2009; Jang, 2010; Fling et al., 2011, 2012). However, most of these studies focused on the topography of TCMF and little is known about development of TCMF. In this study, we hypothesized that development of TCMF would differ according to age and that structural evidence of development of TCMF could be demonstrated by DTT.

In the current study, we attempted to investigate development of TCMF from the corticospinal tract (CST) in the human brain, using DTT.

## MATERIALS AND METHODS

### SUBJECTS

A total of 76 healthy subjects (males: 43, females: 33, mean age: 9.5 years, range: 0–20 years) with no history of neurological, psychiatric, or physical illness were recruited for this study. All participants were volunteers with typical development whose parents had applied to this study. Written informed consent was obtained from the parents of all children. The study was approved by the Institutional Review Board of Yeungnam University hospital.

Subjects were classified into four groups according to age: group A (19 subjects, male: 10, mean age: 2.2 years) – the range of age was from 0 to 5 years, group B (25 subjects, male: 13, mean age: 8.3 years) – the range of age was from 6 to 10 years, group C (20 subjects, male: 15, mean age: 12.7 years) – the range of age was from 11 to 15 years, and group D (12 subjects, male: 5, mean age: 18.4 years) – the range of age was from 16 to 20 years. Each group met the normality on the age.

### DIFFUSION TENSOR TRACTOGRAPHY

A six-channel head coil on a 1.5 T Philips Gyroscan Intera (Philips, Best, The Netherlands) with single-shot echo-planar imaging was used for acquisition of DTI data. For each of the 32 non-collinear diffusion sensitizing gradients, we acquired 60 contiguous slices parallel to the anterior commissure – posterior commissure line. Imaging parameters were as follows: acquisition matrix = 96 × 96; reconstructed to matrix = 128 × 128; field of view = 221 mm × 221 mm; TE = 76 ms; TR = 10,726 ms; parallel imaging reduction factor (SENSE factor) = 2; NEX = 1; EPI factor = 49; *b*-value = 1000 s/mm^2^; and a slice thickness of 2.3 mm (acquired voxel size 1.73 mm × 1.73 mm × 2.3 mm). Removal of eddy current-induced image distortions using affine multi-scale two-dimensional registration was performed using the Oxford centre for functional magnetic resonance imaging of brain software library (FSL; www.fmrib.ox.ac.uk/fsl) (Smith et al., 2004). Signal to noise ratio (SNR_SENSE_) was measured in non-diffusion-weighted images in all subjects, with a mean (SD) of 25.2 (6.2). DTI-Studio software (Johns Hopkins Medical Institute, Baltimore, MD, USA) was used for fiber tracking of the CST (Jiang et al., 2006). Before fiber tracking, DTI data, including the mean diffusivity and fractional anisotropy (FA), were calculated automatically using DTI-Studio software (Jiang et al., 2006). Fiber tracking was based on the fiber assignment continuous tracking algorithm (FACT) and a multiple regions of interest (ROIs) approach. For reconstruction of the entire CST without mixing the adjacent fibers, we selected two ROIs for the CST on the color map (Afifi and Bergman, 2005; Jang, 2011). The first ROI was placed at the upper pons (portion of anterior blue color) on the color map with an axial image. The second ROI was placed on the mid pons (portion of anterior blue color) on the color map with an axial image. Termination criteria used for fiber tracking were FA <0.2 and three tract turning angles of <45, 60, and 75° (Kunimatsu et al., 2004). Incidence was defined as termination of one or more reconstructed fiber into the contralateral cortex via the corpus callosum.

For measurement of inter-observer, random analyses of the data was performed by two evaluators (Kwon HG and Son SM) who were blinded to the other evaluator’s data. The consistency rate of analyses with three tract turning angles by two evaluators were identical for 150 out of 152 hemispheres (98.7%, 45°), 150 out of 152 hemispheres (98.7%, 60°), and 149 out of 152 hemispheres (98.0%, 75°) respectively.

### STATISTICAL ANALYSIS

SPSS software (v.15.0; SPSS, Chicago, IL, USA) was used for data analysis. For comparison with the incidence of TCMF, the chi-square test was performed between the four groups and between the three tract turning angles. The significant level of the *p* value was set at 0.05.

## RESULTS

**FIGURE 1:**
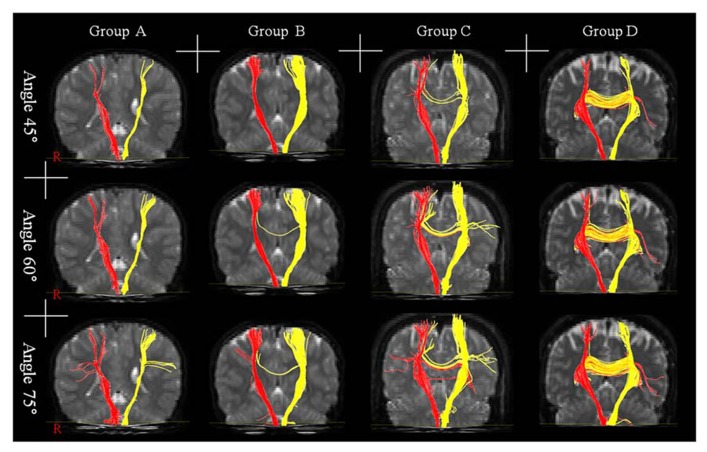
**Results of diffusion tensor tractography for transcallosal motor fiber originating from the corticospinal tract with three angles: group A – the range of age was 0–5 years, group B – the range of age was 6–10 years, group C – the range of age was 11–15 years, and group D – the range of age was 16–20 years**.

A summary of the incidence of TCMF, which originated from the CST, is shown in **Table 1**. No TCMF was found in group A (0–5 years), however, TCMF was found in the other groups, as follows: group B (6–10 years): eight (16%, 45°), 13 (26%, 60°), and 13 (26%, 75°) of 50 hemispheres; group C (11–15 years): 16 (40%, 45°), 24 (60%, 60°), and 25 (63%, 75°) of 40 hemispheres; and group D; 11 (45.83%, 45°), 16 (66.67%, 60°), and 16 (66.67%, 75°) of 24 hemispheres. As a result, incidence of TCMF in groups A and B (under age of 10 years) was lower than in groups C and D (over the age of 10 years) (**Figure 1**).

**Table 1:** Comparison of the incidence of TCMF among four groups.

	45°	60°	75°
Group A (*n* = 19)	0	0	0
Group B (*n* = 25)	8 (16%)	13 (26%)	13 (26%)
Group C (*n* = 20)	16 (40%)	24 (60%)	25 (63%)
Group D (*n* = 12)	11 (45.8%)	16 (66.6%)	16 (66.6%)
*	**0.017**	**0.003**	**0.003**
†	**0.000**	**0.000**	**0.000**
*P* ‡	**0.000**	**0.000**	**0.000**
§	0.053	**0.036**	**0.027**
‖	**0.041**	**0.033**	**0.033**
¶	0.772	0.799	0.875

Incidence (percent as all hemispheres in each group), Bold character: *p* < 0.05. Chi-square test for comparison with incidence of transcallosal fiber among four groups: *, between group A and group B; †, between group A and group C; ‡, between group A and group D; § , between group B and group C; ‖ , between group B and group D; ¶ , between group C and group D.

In comparison with the incidence of difference for TCMF with 45° between the four groups, significant differences were observed between group A and the other three groups [B (*p *= 0.017), C (*p *= 0.000), and D (*p *= 0.000)], and between group B and group D (*p *= 0.041; *p *< 0.05). However, no significant difference was observed between group B and group C (*p *= 0.053), and between group C and group D (*p *= 0.772; *p *> 0.05). Regarding the 60 and 75°, significant differences were observed between group A and the other three groups [B (*p *= 0.003, and 0.003), C (*p *= 0.000, and 0.000), and D (*p *= 0.000, and 0.000)], and between group B and group C (*p *= 0.036, and 0.027), and between group B and group D (*p *= 0.033, and 0.033; *p *< 0.05). However, no significant difference was observed between group C and group D (*p *= 0.799, and 0.879; *p *> 0.05). In addition, no significant differences in the incidence of TCMF were observed between the three tract turning angles (*p *> 0.05).

## DISCUSSION

In the current study, we investigated development of TCMF with age from 0 to 20 years in the human brain. TCMF connects the primary motor cortices of the two hemispheres as neural fiber. However, reliable reconstruction of TCMF between two primary motor cortices with aging was technically difficult; therefore, we were obliged to investigate TCMF derived from the CST. We reconstructed the CST by selection of two ROIs below the corpus callosum: upper and middle pons. With regard to the conditions of fiber tracking for the CST, two conditions, FA and tract turning angle, are important. FA indicates the degree of directionality of microstructures, such as axons, myelin, and microtubules, and has a range of 0 (completely isotropic diffusion) to 1 (completely anisotropic diffusion). Tract turning angles represent the curvature of axonal tracts in a voxel (0–90°). Kunimatsu et al. (2004) reported an optimal FA value of approximately 0.2. However, no optimal degree of tract turning angle has been reported; instead, TCMF tends to be easily changed by the tract turning angle (Lee et al., 2005). Therefore, we reconstructed the CST using the FA value of 0.2 and compared the incidence of TCMF using three different tract turning angles (45, 60, and 75°), which have been adopted popularly in other studies using DTT for the CST (Jang et al., 2009; Son et al., 2009; Kwon et al., 2011).

For comparison of the incidence of TCMF, we divided our subjects into four groups according to age; the two following results were obtained. First, regarding the incidence of TCMF, significant differences were observed between group B and group C, and between group B and group D, with tract turning angles of 60 and 75°. However, no significant differences in any tract turning angles were observed between group C and group D. In addition, the incidence of TCMF in groups A and B (under the age of 10 years) was lower than in groups C and D (over the age of 10 years). Second, regarding the tract turning angle of DTT, in terms of the incidence of TCMF, no significant differences were observed between the three tract turning angles.

Many previous studies have reported on development of IHI (Cohen et al., 1967; Connolly and Stratton, 1968; Wolff et al., 1983; Lazarus and Todor, 1987; Müller et al., 1997; Heinen et al., 1998; Mayston et al., 1999; Gasser et al., 2010). Behavior studies have focused on assessment of associated movements, and have reported that it was normally observed during early childhood and a marked disappearance was observed at the approximate age of 10 years by development of IHI. However, disappearing age of associated movement was a little variable around 10 years [Cohen et al. (1967) – 9 years, Connolly and Stratton (1968) – 5–13 years, Wolff et al. (1983) – 5–8 years, Lazarus and Todor (1987) – 8.5 years]. By contrast, studies using TMS have investigated the development of IHI using the ipsilateral CST (Müller et al., 1997; Heinen et al., 1998). Müller et al. (1997) who investigated development of the ipsilateral CST connection in 50 normal children (range: 3–11 years), found that the incidence of ipsilateral motor evoked potentials (MEP) decreased with aging and the ipsilateral MEP was not observed in children older than 9 years and 9 months of age. Subsequently, using TMS, Heinen et al. (1998) demonstrated the absence of IHI via the corpus callosum in seven children who ranged in age from 4.2 to 5.7 years (Heinen et al., 1998). In 2009, using combined DTI and TMS, Koerte et al. (2009) investigated development of TCMF. They reported significant differences between the two groups [11 children (mean age: 8.4 years; range: 7–11) and 10 adolescents (mean age: 15.6 years, range: 15–17)] in terms of FA on TCMF region of the corpus callosum and duration of the ipsilateral silent period, which is known to depend on activation of IHI. According to the previous studies described above, we can summarize as follows: (1) the age of disappearance of associated movements related to maturation of IHI was reported as approximately 10 years of age. (2) IHI was absent until the age of approximately 5 years. (3) Significant difference in development of TCMF was observed at approximately 10 years of age. Although our method using DTT is different from the research methods used in previous studies, such as behavior, electromyogram, and TMS, the results of previous studies appear to be compatible with those of our study showing that no TCMF was found in group A (0–5 years) and the incidence of TCMF in groups A and B (under age of 10 years) was lower than in groups C and D (over the age of 10 years).

In conclusion, using DTT, we reconstructed visualized TCMF with development and found that the incidence of TCMF differed significantly around the approximate age of 10 years. As a result, we demonstrated structural evidence for development of TCMF in the human brain. We believe that the methodology and results of this study would be helpful to researchers on development of the motor system in the normal human brain and motor recovery mechanisms following brain injury. However, several limitations of this study should be considered (Wiegell et al., 2000; Tuch et al., 2002; Lee et al., 2005; Parker and Alexander, 2005; Yamada, 2009; Yamada et al., 2009). First, we could not show correlation with behavior, such as associated movements; second, due to problems of crossing fibers or partial volume effect, DTI might not reconstruct whole neural fibers, such as TCMF; third, we only investigated the incidence of TCMF without DTT parameters; for example, fiber number and FA; fourth, regarding to the groups according to the age, the number of subjects was not balanced; fifth, the low tesla (1.5), channels (6), and diffusion directions (32) employed in this study are another limitation. Further conduct of combined studies with behavior or electrophysiological study to overcome the limitation of DTI should be encouraged. In addition, conduct of studies on clinical correlation, quantification, reliability, and validity of TCMF would be necessary.

## Conflict of Interest Statement

The authors declare that the research was conducted in the absence of any commercial or financial relationships that could be construed as a potential conflict of interest.
